# Area-level socioeconomic deprivation and cause-specific survival after lung cancer diagnosis: a population-based cohort study in Naples, Italy

**DOI:** 10.3389/fpubh.2026.1959445

**Published:** 2026-10-06

**Authors:** Arianna Scala, Sabrina Cristillo, Ilaria Loperto, Marco Gubitosi, Rosanna Ortolani, Giovanni Improta

**Affiliations:** 1 Department of Public Health, University of Naples Federico II, Naples, Italy; 2 Unit of Epidemiology, Prevention and Cancer Registry - ASL Napoli 1 Centro, Naples, Italy; 3 Department of Law, University of Campania “Luigi Vanvitelli”, Caserta, Italy

**Keywords:** cancer registry, cause-specific survival, competing risks, Cox regression, health inequalities, IDISE, lung cancer, M.A.S.T.E.R.

## Abstract

**Background:**

Lung cancer remains a major public health priority, with survival shaped by clinical, behavioral, socioeconomic, and organizational factors. Socioeconomic disadvantage may affect risk exposure, diagnosis, treatment access, continuity of care, and data completeness.

**Objective:**

To assess the association between area-level socioeconomic deprivation and cause-specific survival in lung cancer and apply the M.A.S.T.E.R. (Methodological Approach for Strategic Transformation, Evaluation, and Redesign) framework to translate epidemiological evidence into organizational design and implementation planning.

**Methods:**

A retrospective population-based registry cohort study within ASL Napoli 1 Centro included incident malignant tumors of the trachea, lung, and bronchi recorded between 2010 and 2023. Cancer Registry data were integrated with the 2021 Socioeconomic Disadvantage Index (IDISE), Hospital Discharge Records, and progressi delle Aziende Sanitarie per la Salute in Italia (PASSI) 2022–2024 contextual smoking prevalence by sex and Primary Health Care District (DSB). Cause-specific survival was analyzed using multivariable Cox regression with district-clustered robust variance; Fine–Gray competing-risk regression was used as sensitivity analysis. M.A.S.T.E.R. comprised six phases: Mapping and contextualization, analysis and diagnosis, strategic design, testing and adaptive execution, embedding and sustainability, and resilience and renewal.

**Results:**

Of 11,162 cases, 10,039 were included in the main analysis, totaling 207,600 person-months. There were 8,514 deaths, including 6,990 cause-specific deaths. In the primary model without contextual smoking prevalence, adjusted hazard ratios (HRs) increased across deprivation quintiles (Q5 vs. Q1: HR 1.11; 95% CI: 1.05–1.18; *p* < 0.001). Adding PASSI smoking prevalence attenuated the estimates (Q5 vs. Q1: HR 1.09; 95% CI: 0.99–1.19; *p* = 0.069); smoking prevalence was not statistically associated with mortality. M.A.S.T.E.R. outputs included a qualitative failure mode and effects analysis (FMEA), a plan, do, study, act (PDSA) protocol with control plan, a completeness dashboard, and plans for updates, audits, and strategic review.

**Conclusions:**

Greater area-level socioeconomic deprivation was associated with higher cause-specific mortality in the primary model; the association was attenuated and no longer statistically significant after adding contextual PASSI smoking prevalence. M.A.S.T.E.R. clarified the transition from epidemiological analysis to candidate interventions for prevention, smoking cessation, early diagnosis, patient navigation, and information integration. Outputs from the final four phases are design-oriented and require prospective implementation and validation.

## 1 Introduction

Lung cancer is a public health priority due to its high incidence, mortality, and the strain it places on healthcare systems. Globally, in 2022, it was the most frequently diagnosed cancer and the leading cause of cancer-related death (1). Tobacco use remains the primary risk factor and accounts for the majority of cases; however, prognosis also depends on the timeliness of diagnosis and access to effective care pathways (2–4). Randomized trials on low-dose computed tomography screening have demonstrated that earlier detection of the disease can reduce cancer-specific mortality in high-risk populations, confirming the central role of stage at diagnosis (5, 6). Despite therapeutic advances, European and Italian data continue to show unsatisfactory survival rates and persistent geographic disparities, including a north-south gradient in Italy; this underscores the value of population-based cancer registries for monitoring incidence, mortality, survival, and equity (7–9).

Survival following a diagnosis of lung cancer results from the interaction of demographic, clinical, behavioral, and contextual factors. Age, sex, histotype, tumor characteristics, and comorbidities are well-established prognostic factors (10). At the same time, socioeconomic disadvantage has repeatedly been associated with poorer survival, particularly in studies using area-level indicators of deprivation (11). The association is complex: deprivation can influence exposure to tobacco smoke, occupational and environmental risks, symptom recognition, access to diagnosis, continuity of care, and the likelihood of receiving appropriate treatments. Population-based evidence indicates that area-level deprivation remains associated with the burden of disease even after adjusting for smoking rates, while mediation analyses suggest that smoking explains a significant, but not complete, portion of the socioeconomic gradient (12, 13).

A recurring methodological challenge in studies based on cancer registries is the lack of individual-level data on smoking. When such data are unavailable, contextual estimates derived from surveillance systems can improve model fit, provided they are interpreted with caution. In Italy, the PASSI system (Progressi delle Aziende Sanitarie per la Salute in Italia) collects local information on behavioral risk factors, including smoking, and thus allows for the estimation of regional prevalence (14). Similarly, deprivation indices allow for the characterization of the socioeconomic context of residence, but require caution regarding the ecological fallacy, since associations observed at the area level do not necessarily correspond to individual mechanisms (15).

Compared with studies that examine individual or contextual determinants separately, this study integrates population-based Cancer Registry data with the IDISE area-level socioeconomic deprivation index and contextual behavioral information from PASSI. The study focused on primary malignant tumors of the trachea, lung, and bronchi recorded within ASL Napoli 1 Centro between 2010 and 2023. The primary epidemiological objective was to assess the association between area-level socioeconomic deprivation and cause-specific mortality using a model adjusted for demographic, clinicopathological, and comorbidity variables. Contextual smoking prevalence was subsequently incorporated in a secondary sensitivity model. The target estimand was the total association between area-level socioeconomic deprivation and cause-specific mortality, rather than an association conditioned on potential downstream clinical factors such as stage at diagnosis or treatment received.

To move beyond a purely descriptive analysis and make explicit the transition from epidemiological evidence to organizational design, the analysis and subsequent operational translation of the results were organized according to the M.A.S.T.E.R. framework (Methodological Approach for Strategic Transformation, Evaluation, and Redesign). The method integrates the visual clarity of A3 thinking, the analytical rigor of DMAIC, and the iterative logic of PDCA across six phases: Mapping and contextualization, analysis and diagnosis, strategic design, testing and adaptive execution, embedding and sustainability, and resilience and renewal. Recent applications of the framework have focused on improving the timeliness of surgery for older adults with proximal femoral fractures, implementing a fast-track pathway for total hip replacement, and reducing the length of hospital stay following laparoscopic appendectomy (16–18).

In this study, the first two phases of M.A.S.T.E.R. involve the development of an integrated information system and the analysis of observed epidemiological results, while the four subsequent phases translate the evidence into strategic actions, prospective testing protocols, standardization tools, and a plan for periodic review. This distinction is essential: the study does not demonstrate the effectiveness of an already implemented M.A.S.T.E.R. intervention, but rather proposes a structured methodological approach to local cancer surveillance, to be subjected to future prospective and multicenter evaluation.

## 2 Materials and methods

A retrospective population-based registry cohort study was conducted within ASL Napoli 1 Centro. The methodological framework follows the six phases of the M.A.S.T.E.R. model; the correspondence with PDCA, DMAIC, the tools used, and the expected outcomes is shown in Table 1.

**Table 1:** Operational integration of the M.A.S.T.E.R. framework in this study.

M.A.S.T.E.R. phase	PDCA	DMAIC	Tools applied or prepared	Outputs of this study
Mapping and contextualization	Plan	Define	A3 problem statement; SIPOC; stakeholder map; description of information flows	Problem, purpose, population, data sources, system boundaries, stakeholders, and information flow architecture
Analysis and diagnosis	Do	Measure-analyze	Pareto chart; causal analysis based on the 5 Whys; Multivariable Cox regression; Fine-Gray model as a sensitivity analysis; interaction analysis	Morphological baseline, prognostic associations, directional consistency across competing-risk analyses, and plausible mechanisms
Strategic design	Plan/Do	Improve	Qualitative FMEA	Proposed priority failure modes and candidate strategic actions
Testing and adaptive execution	Check	Control	PDSA; Control plan	Prospective protocol for piloting, monitoring, and adaptation based on KPIs
Embedding and sustainability	Act	Consolidated control	Dashboard; completeness checklist; training plan; Kamishibai/LPA audit	Standardization of data and processes, responsibilities, monitoring, and maintenance
Resilience and renewal	New cycle	New iteration	Annual strategic review; completeness audit; updating of data and estimates; drift analysis; external validation	Monitoring of stability, adaptation, transferability, and the next iteration of M.A.S.T.E.R.

### 2.1 Mapping and contextualization

The Mapping and contextualization phase defined the problem, the scope of the study, the stakeholders, and the information flows through the formulation of the A3 problem statement, the SIPOC diagram, stakeholder mapping, and the description of information flows. The A3 problem was formulated as follows: the presence of a possible socioeconomic gradient in lung cancer survival that cannot be fully explained by clinical and demographic data from the Cancer Registry alone. The aim was to integrate complementary routine data sources and produce operational recommendations tailored to specific regions.

The study population includes all new cases of primary malignant tumors of the trachea, lung, and bronchi, identified using ICD-10 codes C33 and C34 and recorded between January 1, 2010, and December 31, 2023. In the primary analysis, records with a non-positive observed survival time ( ≤ 0 months) were excluded. This operational criterion excluded 666 records. DCO status could not be separately identified in the analytical dataset used for the survival models. Consequently, the number of DCO cases and their overlap with records having non-positive survival time could not be determined separately, and DCO status was not treated as synonymous with non-positive survival time. As a sensitivity analysis, records with non-positive survival time were retained by assigning a minimal follow-up time of 0.1 months, after which the primary multivariable cause-specific Cox model was refitted using the same covariates and DSB-clustered robust variance. When multiple records referred to the same patient, only the first tumor in chronological order was included to preserve the statistical independence of the observations.

Residence at the time of diagnosis was determined using a symmetric temporal cascade record-linking procedure with historical municipal registry records for the period 2010–2023. This strategy was adopted to maximize georeferencing and reduce missing information regarding the context of residence. Cases residing in the island municipalities of Capri and Anacapri, for which a specific neighborhood was not available, were mapped separately using ISTAT codes. Based on the standardized neighborhood, each patient was assigned to the geographically competent Primary health care district (DSB).

Contextual socioeconomic status was measured using the 2021 Socioeconomic Disadvantage Index (IDISE) (19). The continuous scores were divided into territorial quintiles Q1–Q5: Q1 identifies the least deprived areas and was used as a reference, while Q5 identifies the areas with the greatest social disadvantage. Because the 2021 IDISE was applied to patients diagnosed throughout 2010–2023, it should be interpreted as a single-time-point area-level socioeconomic classification rather than as a contemporaneous measure of socioeconomic conditions at the time of each cancer diagnosis. This temporal mismatch may have introduced exposure misclassification, particularly for patients diagnosed in the earlier years of the study period. No assumption of temporal invariance was made. Rather, IDISE 2021 was treated as a single-time-point area-level classification whose correspondence with socioeconomic conditions in earlier diagnostic years may be imperfect.

Demographic and oncological variables were obtained from the Cancer Registry and included sex, age at diagnosis, tumor site, morphology/histotype, and histological grade according to ICD-O-3. Comorbidity information was obtained by record linkage with a precomputed Charlson Comorbidity Index (CCI) dataset derived from Hospital Discharge Records available for 2014–2023. The CCI variables were linked to the cancer cohort through the individual identifier and categorized as CCI 0, CCI 1, and CCI 2+. Because the CCI was obtained from a previously constructed administrative dataset, the study-level analytical file did not contain the underlying hospitalization dates or a patient-specific look-back window relative to cancer diagnosis. Consequently, the temporal ordering of all hospital diagnoses contributing to the CCI could not be independently reconstructed within the present analysis. Patients without a match in the Charlson dataset were retained in a separate “No linked CCI record/unknown” category. This category indicates absence of an available linked CCI record and should not be interpreted as a clinical comorbidity category or as evidence of absence of hospitalization. Since the Cancer Registry did not contain individual smoking histories, an ecological adjustment based on PASSI was applied. Microdata for the years 2022, 2023, and 2024, along with the corresponding supplementary forms, were extracted and integrated. The dichotomous variable “current smoker” was defined, excluding non-respondents, and specific percentage prevalences were calculated for the 22 mutually exclusive combinations of sex and DSB present in the ASL Napoli 1 Centro. Prevalence was linked to the cancer cohort as a continuous covariate representing contextual smoking prevalence. PASSI was not used to attribute individual exposure but rather as a territorial proxy. Because PASSI estimates were derived from surveillance data collected in 2022–2024, whereas cancer diagnoses occurred between 2010 and 2023, this covariate should be interpreted exclusively as an ecological indicator of the recent geographic distribution of smoking rather than as a temporally aligned measure of smoking exposure throughout the cohort. Its inclusion was exploratory and intended to account, albeit imperfectly, for spatial variation in smoking prevalence in the absence of individual smoking histories. Similarly, no assumption was made that PASSI 2022–2024 smoking prevalence remained constant throughout the 2010–2023 cohort period. This phase was completed by mapping the stakeholders involved in the production, integration, interpretation, and use of the data. The following were considered: the Cancer Registry, institutional epidemiology, Primary Health Care Districts, general practitioners, smoking cessation services, the operational units of pulmonology, radiology, pathological anatomy, and oncology, strategic management, institutional information systems, and the resident population.

Stakeholders were qualitatively classified based on four dimensions: role within the system, level of interest in the study's outcomes, ability to influence organizational decisions, and potential contribution to the use of the results. Levels of interest and influence were categorized as high, medium-high, medium, and medium-low. Influence was assessed by considering data availability, decision-making responsibility, the ability to initiate local interventions, and involvement in diagnostic and care pathways.

The SIPOC diagram was used to represent data providers, information inputs, key integration and analysis activities, outputs, and recipients of the findings. At the same time, stakeholder mapping made it possible to identify the stakeholders to be involved in the subsequent phases of planning, implementation, and monitoring of the actions.

### 2.2 Analysis and diagnosis

The analysis and diagnosis phase integrated quality-improvement tools with epidemiological methods. The Pareto chart was used to rank the main morphological categories according to their frequency and to identify those contributing most substantially to the overall case distribution. Subsequently, the observed findings were organized using a causal-analysis matrix inspired by the 5 Whys technique, with the aim of linking the observed survival patterns to potential behavioral, diagnostic, informational, and organizational mechanisms. This tool was used for interpretive purposes only, explicitly distinguishing directly observed associations, available contextual proxies, and plausible but unmeasured mechanisms. The epidemiological analysis included multivariable Cox regression, Fine–Gray competing-risk regression, and an exploratory analysis of the interaction between socioeconomic deprivation and sex. Baseline characteristics of the final analytical cohort were summarized overall and by IDISE deprivation quintile. Categorical variables were reported as counts and percentages, age and contextual smoking prevalence as mean and standard deviation (SD), and follow-up duration as median and interquartile range (IQR). For descriptive purposes, year of diagnosis was grouped into three periods: 2010–2014, 2015–2019, and 2020–2023. Missingness in the variables reported in the baseline table was also assessed.

Follow-up was calculated in months from the date of incidence until the date of death or censoring. The primary endpoint was cause-specific survival (CSS). A strict coding algorithm was used to define cause-specific deaths: among deceased patients, an event was classified as lung-cancer-specific when the recorded cause-of-death field was coded as ICD-10 C33–C34. When the ICD-10 cause-of-death field was unavailable, ICD-9 code 162 was used as a fallback. Deaths that did not meet either criterion were not classified as lung-cancer-specific deaths. In the cause-specific Cox analysis, these deaths were censored at the date of death, whereas in the Fine–Gray analysis they were classified as competing events. Accordingly, deaths assigned to other causes, including causes not meeting the predefined lung-cancer coding criteria, were not attributed to lung cancer. Uncertain or ill-defined causes were therefore handled conservatively as non-lung-cancer deaths rather than being reassigned to the cause-specific endpoint. The association between IDISE quintiles and cause-specific mortality was estimated using multivariable semiparametric Cox models, with hazard ratios (HRs) and 95% confidence intervals. The target estimand was the total association between area-level socioeconomic deprivation and cause-specific mortality. The analysis was not designed to estimate an association independent of stage at diagnosis or treatment received. These variables were not available in the analytical dataset and may also represent downstream pathways through which socioeconomic disadvantage influences survival; therefore, their absence precludes assessment of the mechanisms underlying the observed association rather than allowing those mechanisms to be excluded. The primary model included IDISE quintile, sex, age, morphology/histotype, topography, and CCI. Contextual smoking prevalence derived from PASSI 2022–2024 was subsequently added as a secondary sensitivity model to assess whether accounting for the recent geographic distribution of smoking materially changed the deprivation estimates. PASSI smoking prevalence was modeled as a continuous covariate, with the HR expressed per one-percentage-point increase. This parsimonious specification was chosen because PASSI represented an ecological contextual adjustment based on a limited number of sex-by-DSB strata rather than an individual-level exposure. Categorization would have required arbitrary cut-points and further reduced the information available within contextual strata, while more flexible nonlinear specifications would have increased model complexity in an already limited cluster-level structure. The linear specification should therefore be regarded as an exploratory modeling choice rather than evidence that the association between contextual smoking prevalence and mortality is necessarily linear. Calendar period was additionally examined in a sensitivity model by adding two indicator terms (2015–2019 and 2020–2023, with 2010–2014 as reference) to the primary Cox specification. A second temporally restricted sensitivity analysis was conducted among patients diagnosed in 2019–2023. Given its ecological nature and temporal mismatch with the cancer cohort, contextual smoking prevalence was not interpreted as a measure of individual or contemporaneous smoking exposure, nor as formal evidence of confounding or mediation. For the Cox models, robust variance estimates clustered at the Primary Health Care District (DSB) level were used to account for within-district correlation. The final analytical model included 10 DSB clusters. Because inference based on cluster-robust sandwich variance may be sensitive when the number of clusters is small, the primary model was compared with the corresponding Cox model fitted using conventional model-based variance estimates and with a finite-sample corrected cluster-robust analysis. For the latter, the cluster-robust covariance matrix was multiplied by [G/(G−1)] × [(N−1)/(N–p)], where G = 10 clusters, *N* is the sample size, and *p* is the number of model coefficients; confidence intervals and *p*-values were then based on a t distribution with G−1 = 9 degrees of freedom. The distribution of patients and cause-specific deaths across DSB clusters was also examined. The proportional-hazards assumption was assessed using Schoenfeld-residual diagnostics based on the association between residuals and log follow-up time, with both term-specific and global tests. Because the IDISE terms showed no evidence of non-proportionality, no time-varying IDISE effects were introduced; departures detected for some adjustment covariates are reported as a limitation.

The Fine-Gray model was applied as a sensitivity analysis with respect to the presence of competing risks, considering deaths from causes other than tracheal, lung, or bronchial cancer as competing events. The cause-specific Cox and Fine–Gray models address different estimands. The Cox model estimates the relative instantaneous hazard of cause-specific death among individuals who remain event-free, treating deaths from other causes as censoring events, whereas the Fine–Gray model estimates the subdistribution hazard associated with the cumulative incidence of cause-specific death in the presence of competing mortality. Therefore, HRs and subdistribution hazard ratios (sHRs) were interpreted as complementary rather than directly interchangeable measures.

An exploratory deprivation-by-sex interaction model was fitted by including product terms between IDISE quintile and sex in the primary cause-specific Cox model, with Q1 and female sex as the reference categories. The interaction terms therefore quantify the multiplicative modification of the deprivation hazard ratio in males relative to females. Effect modification was evaluated using the term-specific coefficients, hazard ratios, 95% confidence intervals, and *p*-values, together with a joint four-degree-of-freedom Wald test of all IDISE-by-sex interaction terms. Statistical significance was set at a two-sided *p*-value < 0.05. Data processing, management, and the original epidemiological analyses were performed using STATA 19BE. Additional revision-stage sensitivity analyses and diagnostic checks were independently reproduced from the analytical dataset using Python 3.13 and statsmodels 0.14.6.

### 2.3 Strategic design

The Strategic design phase translated epidemiological findings, identified data gaps, and hypothesis-generating mechanisms into candidate intervention options using a simplified qualitative FMEA of the prevention-diagnosis-care pathway. This tool made it possible to identify the main modes of failure potentially associated with survival disparities, describe their effects, evaluate the available evidence, and define possible corrective actions.

Priorities were assigned qualitatively, into the categories of high and medium-high, considering the potential impact on diagnostic timeliness, continuity of care, completeness of information, and equity of access, as well as consistency with the results, the ability to reduce inequalities, organizational feasibility, data availability, dependence on external resources, monitorability, and integration into existing pathways.

The options included: intensified smoking prevention and cessation efforts in the most deprived DSBs with the highest prevalence of smoking; targeted outreach and recruitment for future low-dose CT screening programs; patient navigation and active outreach; greater integration between primary care, diagnostics, and oncology; systematic recording of stage, treatment, and individual smoking history; expansion of record linkage to reduce the “No linked CCI record/unknown” category.

The FMEA was intended solely for planning and interpretive purposes. No interventions were implemented, no patients were assigned, and no prospective outcomes were measured; therefore, the failure modes and proposed actions do not represent proven causal relationships or evidence of effectiveness.

### 2.4 Testing and adaptive execution

In this retrospective application, the Testing and adaptive execution phase was operationalized through the development of a prospective PDSA protocol and a control plan. The proposed pilot involves selecting one or more DSBs based on the level of deprivation, the prevalence of smoking in the area, critical issues identified in the KPIs, and organizational feasibility, followed by baseline measurement, the modular implementation of smoking cessation interventions, patient navigation, and improved data linkage, periodic monitoring of KPIs, and adjustment of actions.

The Control Plan defines, for each KPI, the calculation method, data source, measurement frequency, person responsible, and response protocol. The indicators pertain to the completeness of staging and treatment, documentation of smoking history, referral to smoking cessation services, time from suspicion to diagnosis and from diagnosis to treatment, adherence to active follow-up calls, loss to follow-up, and differences between deprivation quintiles and DSB. Numerical targets will be defined after the prospective baseline survey and have not been assigned retrospectively to the available data.

### 2.5 Embedding and sustainability

The embedding and sustainability phase defined the tools needed to incorporate improvements into routine practice: a district dashboard, a case completeness checklist, a training plan, and the Kamishibai/Layered Process Audit (LPA). The dashboard integrates indicators for outcomes, processes, equity, data quality, and sustainability, stratified—where applicable—by sex, DSB, and IDISE quintile. For each domain, indicators, data sources, update frequency, responsible parties, and actions to be taken in the event of deviations were defined. The checklist was designed to verify the presence of essential information before a case is closed. The training plan covers the correct recording of data, the use of the dashboard, and the management of corrective actions. Kamishibai/LPA audits have been established as brief, standardized periodic checks on the application of procedures.

Sustainability was not measured during the retrospective period; the tools described therefore represent a governance proposal to be submitted for future implementation and prospective evaluation.

### 2.6 Resilience and renewal

The Resilience and renewal phase established a periodic plan for updates and strategic review aimed at verifying, over time, the stability of the data, the observed associations, and the organizational priorities. The plan includes the annual updating of data flows from the Cancer Registry, PASSI, and SDO; the use of the most recent available version of IDISE; periodic completeness audits; a re-estimation of the Cox and Fine-Gray models; and an analysis of temporal drift. The drift analysis is designed to identify any changes in the geographic distribution of deprivation and smoking, in the composition of the case series, in the completeness of the variables, and in the strength of prognostic associations. The cycle also includes comparison with other local health authorities (ASLs) or registries, when available, and multicenter validation of the transferability of the results.

Any future individual predictive models will need to be developed using data enriched with stage, treatment, performance status, individual smoking history, and indicators of the care pathway, and will need to be evaluated in terms of discrimination, calibration, equity, and external validity. This phase is exploratory in nature: prospective updating, multicenter validation, and the development of predictive models were not performed within the retrospective cohort.

## 3 Results

The mapping and contextualization and analysis and diagnosis phases produced empirical results derived from the cohort. The strategic design, testing and adaptive execution, embedding and sustainability, and resilience and renewal phases produced design and governance outputs; since no interventions were implemented during the observation period, no outcome improvements attributable to the framework are reported. The complete operational tools for the six phases are presented in the Supplementary material (Supplementary Tools S1–S6).

### 3.1 Results of the mapping and contextualization phase

The mapping and contextualization phase produced four complementary methodological outputs: the formulation of the A3 problem statement, the representation of the system using the SIPOC diagram, the description of information flows, and the stakeholder mapping.

The A3 problem statement identified as the central issue the presence of a possible socioeconomic gradient in lung cancer survival that cannot be adequately characterized using the demographic and clinicopathological information available from the Cancer registry alone. The resulting objective was to integrate complementary routine data sources to improve the interpretation of the observed inequalities and produce operational recommendations tailored to specific regions. The SIPOC diagram represented the study's information architecture and highlighted a system composed of complementary but not originally integrated sources. The Cancer registry provided data on incidence, morphology, topography, date of diagnosis, and follow-up; the historical population registry enabled georeferencing; IDISE described the socioeconomic context; the SDOs allowed for the estimation of comorbidities; and PASSI provided the contextual prevalence of smoking. The description of the information flows highlighted the pathway that, starting from the individual sources, led through the procedures of record linkage, geocoding, and integration to the construction of the final analytical dataset. The primary recipients of the evidence produced were the institutional epidemiology department, the DSBs, clinical services, and strategic management. The complete SIPOC diagram is provided in the Supplementary material (Supplementary Tool S1, Supplementary Table S1).

The stakeholder mapping revealed that the Cancer Registry, organizational epidemiology, the DSBs, and strategic management are the actors characterized by both high interest and high influence. These entities play a central role both in the availability and interpretation of data and in translating evidence into organizational and regional decisions.

General practitioners, smoking cessation services, and clinical-diagnostic services demonstrate a high level of interest but a predominantly moderate capacity for influence, as their contribution primarily involves identifying at-risk individuals, initiating preventive interventions, guiding diagnosis, and managing patient care. The resident population is the primary target of these actions, with a high level of interest but a limited direct capacity to influence organizational decisions. A complete mapping of stakeholders is provided in the Supplementary material (Supplementary Tool S1, Supplementary Table S2).

Defining the system boundaries and data sources made it possible to identify the target population and establish the selection process for the analytical cohort. Between 2010 and 2023, the Cancer Registry identified 11,162 new cases of primary malignant tumors of the trachea, lung, and bronchi. Of the 11,162 recorded cases, 666 had a non-positive observed survival time (≤ 0 months) and were excluded from the primary survival analysis, leaving 10,496 cases after application of the survival-time criterion. Among these, 457 cases did not have an assignable IDISE deprivation quintile and therefore did not enter the deprivation models, yielding a final analytical cohort of 10,039 patients. The total time at risk was 207,600 person-months. During follow-up, 8,514 deaths were recorded, including 6,990 cause-specific deaths attributed to lung cancer. Baseline characteristics of the final analytical cohort stratified by IDISE deprivation quintile are presented in Table 2, while detailed distributions of tumor morphology and topography across deprivation quintiles are provided in the Supplementary material (Supplementary Tool S2, Supplementary Table S3). The deprivation groups differed in several demographic and contextual characteristics. Patients in Q1 were older on average than those in the other quintiles (mean age 71.4 years in Q1 vs. 68.0 years in Q5) and had a lower proportion of males (58.9% vs. 68.4% in Q5). The proportion of cause-specific deaths increased modestly from 68.0% in Q1 to 71.3% in Q5. Diagnosis-period distributions were relatively similar across quintiles, while morphology, topography, and CCI/data-availability categories showed only modest variation. Contextual smoking prevalence was lowest in Q1 (mean 22.8%) and highest in Q5 (33.1%). Median follow-up was broadly comparable across deprivation groups, ranging from 8.1 to 9.0 months.

**Table 2:** Baseline characteristics of the final analytical cohort according to IDISE 2021 deprivation quintile.

Characteristic	Q1	Q2	Q3	Q4	Q5
Patients, *n*	2,584	2,214	1,673	2,151	1,417
Cause-specific deaths, *n* (%)	1,756 (68.0)	1,542 (69.6)	1,160 (69.3)	1,522 (70.8)	1,010 (71.3)
Age, mean (SD), years	71.4 (9.8)	68.9 (10.5)	67.9 (10.2)	67.3 (10.7)	68.0 (10.5)
Male sex, *n* (%)	1,522 (58.9)	1,447 (65.4)	1,155 (69.0)	1,427 (66.3)	969 (68.4)
Diagnosis period, *n* (%)					
2010–2014	839 (32.5)	719 (32.5)	493 (29.5)	650 (30.2)	454 (32.0)
2015–2019	964 (37.3)	796 (36.0)	656 (39.2)	803 (37.3)	497 (35.1)
2020–2023	781 (30.2)	699 (31.6)	524 (31.3)	698 (32.5)	466 (32.9)
CCI/data availability, *n* (%)					
CCI 0	208 (8.0)	162 (7.3)	106 (6.3)	135 (6.3)	76 (5.4)
CCI 1	74 (2.9)	63 (2.8)	50 (3.0)	58 (2.7)	46 (3.2)
CCI 2+	1,617 (62.6)	1,465 (66.2)	1,090 (65.2)	1,437 (66.8)	940 (66.3)
No linked CCI record/unknown	685 (26.5)	524 (23.7)	427 (25.5)	521 (24.2)	355 (25.1)
Contextual smoking prevalence, mean (SD), %	22.8 (5.4)	30.5 (5.3)	32.9 (4.0)	30.8 (4.6)	33.1 (4.1)
Follow-up, median (IQR), months	8.8 (2.3–26.3)	9.0 (2.2–25.6)	8.1 (2.1–23.3)	8.2 (2.1–24.9)	8.3 (2.2–23.0)

Values are *n* (%) unless otherwise indicated. Percentages are calculated within deprivation quintiles. Contextual smoking prevalence is an ecological sex- and DSB-specific measure derived from PASSI surveillance and does not represent individual smoking exposure. After application of the survival-time criterion, 457 patients without an assignable IDISE quintile were excluded from the deprivation analysis. No missing values remained for the variables reported in the analytical cohort. CCI, Charlson Comorbidity Index; DSB, Primary Health Care District; IQR, interquartile range; PASSI, Progressi delle Aziende Sanitarie per la Salute in Italia; SD, standard deviation.

### 3.2 Results of the analysis and diagnosis phase

Among the 11,162 new cases recorded overall during the 2010–2023 period, adenocarcinomas represented the most common morphological category (39.0%), followed by other/unspecified (33.8%), squamous cell carcinomas (15.5%), neuroendocrine tumors (11.6%), and sarcomas (0.1%). The Pareto chart identified adenocarcinoma and the “other/unspecified” category as the two main components of the morphological distribution, together accounting for 72.8% of the recorded cases. In particular, the high frequency of the unspecified category was considered a possible indication of incomplete diagnostic characterization or heterogeneity in the data, to be further investigated in subsequent phases of the framework. The Pareto chart is presented in the Supplementary material (Supplementary Tool S2, Supplementary Figure S1).

The adjusted cause-specific survival curves, shown in Figure 1, revealed a gradual but modest divergence across the deprivation quintiles, with slightly more favorable outcomes among residents of less deprived areas and less favorable trends in the higher quintiles.

**Figure 1:**
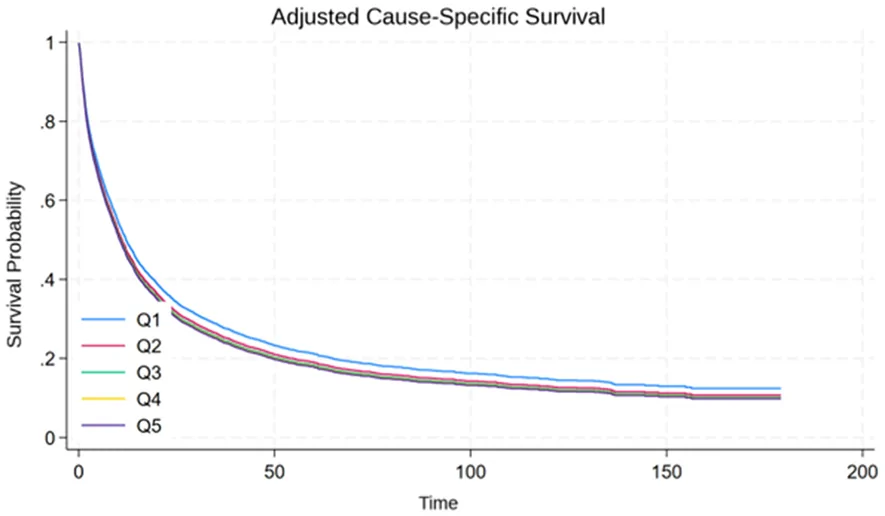
Cause-specific survival adjusted for IDISE quintile. The curves are derived from the primary Cox model adjusted for sex, age, morphology/histotype, topography, and comorbidity.

In the primary multivariable Cox model without PASSI contextual smoking prevalence (Table 3), adjusted HR point estimates increased progressively across deprivation quintiles. Compared with Q1, the HRs were 1.07 for Q2 (95% CI: 1.01–1.13; *p* = 0.020), 1.10 for Q3 (95% CI: 1.03–1.16; *p* = 0.002), 1.11 for Q4 (95% CI: 1.03–1.18; *p* = 0.005), and 1.11 for Q5 (95% CI: 1.05–1.18; *p* < 0.001).

**Table 3:** Association between area-level socioeconomic deprivation and cause-specific mortality in the primary and PASSI-adjusted Cox models.

Variable	Primary model without PASSI HR (95% CI)	*p*	PASSI-adjusted model HR (95% CI)	*p*
IDISE Q2 vs. Q1	1.070 (1.011–1.133)	0.020	1.050 (0.978–1.127)	0.175
IDISE Q3 vs. Q1	1.096 (1.034–1.161)	0.002	1.070 (0.985–1.161)	0.110
IDISE Q4 vs. Q1	1.105 (1.031–1.185)	0.005	1.085 (0.987–1.191)	0.090
IDISE Q5 vs. Q1	1.112 (1.050–1.179)	< 0.001	1.086 (0.994–1.186)	0.069
Contextual smoking prevalence	—	—	1.002 (0.998–1.007)	0.274

When contextual PASSI smoking prevalence was added as a secondary sensitivity adjustment, the deprivation estimates were modestly attenuated: HR 1.05 for Q2 (95% CI: 0.98–1.13; *p* = 0.175), 1.07 for Q3 (95% CI: 0.98–1.16; *p* = 0.110), 1.08 for Q4 (95% CI: 0.99–1.19; *p* = 0.090), and 1.09 for Q5 (95% CI: 0.99–1.19; *p* = 0.069). Contextual smoking prevalence itself was not statistically associated with cause-specific mortality (HR 1.002 per one-percentage-point increase; 95% CI: 0.998–1.007; *p* = 0.274). Because PASSI represents an ecological and temporally non-contemporaneous measure, the attenuation of the deprivation estimates should not be interpreted as evidence of individual-level confounding or mediation by smoking.

Additional sensitivity analyses addressed temporal alignment, CCI uncertainty, and small-cluster inference. After adjustment for diagnostic period (2010–2014 reference; 2015–2019 and 2020–2023 indicators), the deprivation estimates were essentially unchanged: Q2 HR 1.071 (95% CI 1.012–1.135; *p* = 0.018), Q3 HR 1.091 (95% CI 1.034–1.151; *p* = 0.002), Q4 HR 1.103 (95% CI 1.027–1.184; *p* = 0.007), and Q5 HR 1.117 (95% CI 1.051–1.187; *p* < 0.001). In the analysis restricted to diagnoses in 2019–2023 (*n* = 3,940; 2,297 cause-specific deaths), point estimates remained above unity, although precision was lower: Q2 HR 1.087 (95% CI 0.914–1.293; *p* = 0.345), Q3 HR 1.224 (95% CI 1.046–1.432; *p* = 0.012), Q4 HR 1.237 (95% CI 1.031–1.483; *p* = 0.022), and Q5 HR 1.124 (95% CI 0.970–1.303; *p* = 0.121). A model omitting CCI produced closely comparable deprivation estimates (Q5 vs. Q1 HR 1.106; 95% CI 1.054–1.161; *p* < 0.001), indicating that the main deprivation association was not dependent on CCI adjustment. With finite-sample correction of the DSB-clustered covariance and t-based inference with 9 degrees of freedom, the corresponding estimates remained statistically significant for Q2–Q5; for Q5 vs. Q1, HR 1.112 (95% CI 1.040–1.189; *p* = 0.006). Complete results are reported in Supplementary Tables S11–S15. Schoenfeld-residual diagnostics showed no evidence of non-proportionality for the IDISE terms (Q2 *p* = 0.621; Q3 *p* = 0.334; Q4 *p* = 0.923; Q5 *p* = 0.279), whereas the global model diagnostic was significant (*p* < 0.001), indicating non-proportionality for one or more adjustment covariates. Complete proportional-hazards diagnostics are reported in Supplementary Table S16.

The primary Cox model was based on 10 DSB clusters. Comparison with the corresponding model using conventional model-based variance estimates showed identical point estimates and a broadly consistent deprivation pattern. Without cluster-robust variance, the HRs for Q2–Q5 vs. Q1 were 1.07 (95% CI: 1.00–1.15; *p* = 0.053), 1.10 (95% CI: 1.02–1.18; *p* = 0.017), 1.11 (95% CI: 1.03–1.18; *p* = 0.005), and 1.11 (95% CI: 1.03–1.20; *p* = 0.008), respectively. Thus, the direction and statistical significance of the estimates for Q3–Q5 were preserved across variance specifications, whereas inference for Q2 was more sensitive to the variance estimator. In a further sensitivity analysis addressing the exclusion of non-positive survival times, the 666 records with survival time ≤ 0 months were retained by assigning a minimal follow-up time of 0.1 months. The fully adjusted cause-specific Cox model then included 10,663 patients and 7,607 cause-specific deaths. The deprivation estimates remained similar to those observed in the primary analysis: HR 1.083 for Q2 vs. Q1 (95% CI: 1.033–1.135; *p* = 0.001), 1.099 for Q3 (95% CI: 1.035–1.167; *p* = 0.002), 1.115 for Q4 (95% CI: 1.024–1.214; *p* = 0.013), and 1.117 for Q5 (95% CI: 1.057–1.180; *p* < 0.001). These findings indicate that the deprivation estimates were not materially altered by retaining records with non-positive survival time using a minimal positive follow-up value. The associations between the main demographic, morphological, topographic, and clinical covariates are presented in Table 4. Male sex was associated with an approximately 20% higher cause-specific mortality hazard compared with female sex. Age was associated with an approximately 1.2% increase in hazard per additional year. Compared with squamous cell carcinomas, adenocarcinomas had a comparable risk, while neuroendocrine tumors and the “other/unspecified” category had a worse prognosis. Sarcomas showed an estimated risk greater than one but not statistically significant, likely due to the extremely small sample size.

**Table 4:** Hazard ratios for the main covariates in the primary multivariable Cox model for cause-specific survival.

Scope	Variable/category	Reference category/scale	HR	95% CI	*p*-value
Demographics	Male	Female	1.204	1.152–1.258	< 0.001
Demographics	Age at diagnosis	Per 1-year increase	1.012	1.008–1.017	< 0.001
Histotype/morphology	Adenocarcinoma	Squamous cell carcinoma	0.959	0.900–1.023	0.208
Histotype/morphology	Neuroendocrine tumors	Squamous cell carcinoma	1.443	1.354–1.539	< 0.001
Histotype/morphology	Sarcomas	Squamous cell carcinoma	1.369	0.720–2.606	0.338
Histotype/morphology	Other/unspecified	Squamous cell carcinoma	1.731	1.563–1.917	< 0.001
Topography	Upper lobe	Trachea/main bronchi	0.559	0.501–0.622	< 0.001
Topography	Middle lobe	Trachea/main bronchi	0.628	0.560–0.704	< 0.001
Topography	Lower lobe	Trachea/main bronchi	0.510	0.473–0.551	< 0.001
Topography	Overlapping lesion	Trachea/main bronchi	0.815	0.697–0.953	0.010
Topography	Unspecified	Trachea/main bronchi	0.839	0.764–0.921	< 0.001
Comorbidity	Charlson index 1	Charlson index 0	0.908	0.775–1.063	0.229
Comorbidity	Charlson index 2+	Charlson index 0	1.096	1.013–1.185	0.023
Data availability/comorbidity	No linked CCI record/Unknown	Charlson index 0	1.770	1.560–2.008	< 0.001

Compared with the trachea and main bronchi, known lobar sites were associated with a more favorable prognosis: a risk reduction of between approximately 37% and 49%. Overlapping lesions and unspecified sites also showed HRs below 1, although the magnitude of the association was less pronounced than for known lobar sites. CCI 1 was not significantly associated with the outcome, whereas CCI 2+ was associated with a modest increase in cause-specific mortality. The “No linked CCI record/unknown” category showed the largest positive association among the CCI/data-availability categories (HR 1.77). However, because this category identifies patients without an available linked CCI record rather than a defined clinical condition, this association should not be interpreted as evidence of greater comorbidity burden, frailty, absence of hospitalization, or fragmented care.

The graphical representation of the Cox model estimates, shown in Figure 2, confirmed the direction and magnitude of the associations described in Table 4. In particular, male sex, older age, neuroendocrine tumors, unspecified morphology, severe comorbidity, and the “No linked CCI record/unknown” data-availability category were associated with higher cause-specific mortality. Conversely, known lobar locations showed a more favorable prognostic profile than the trachea/main bronchus reference category.

**Figure 2:**
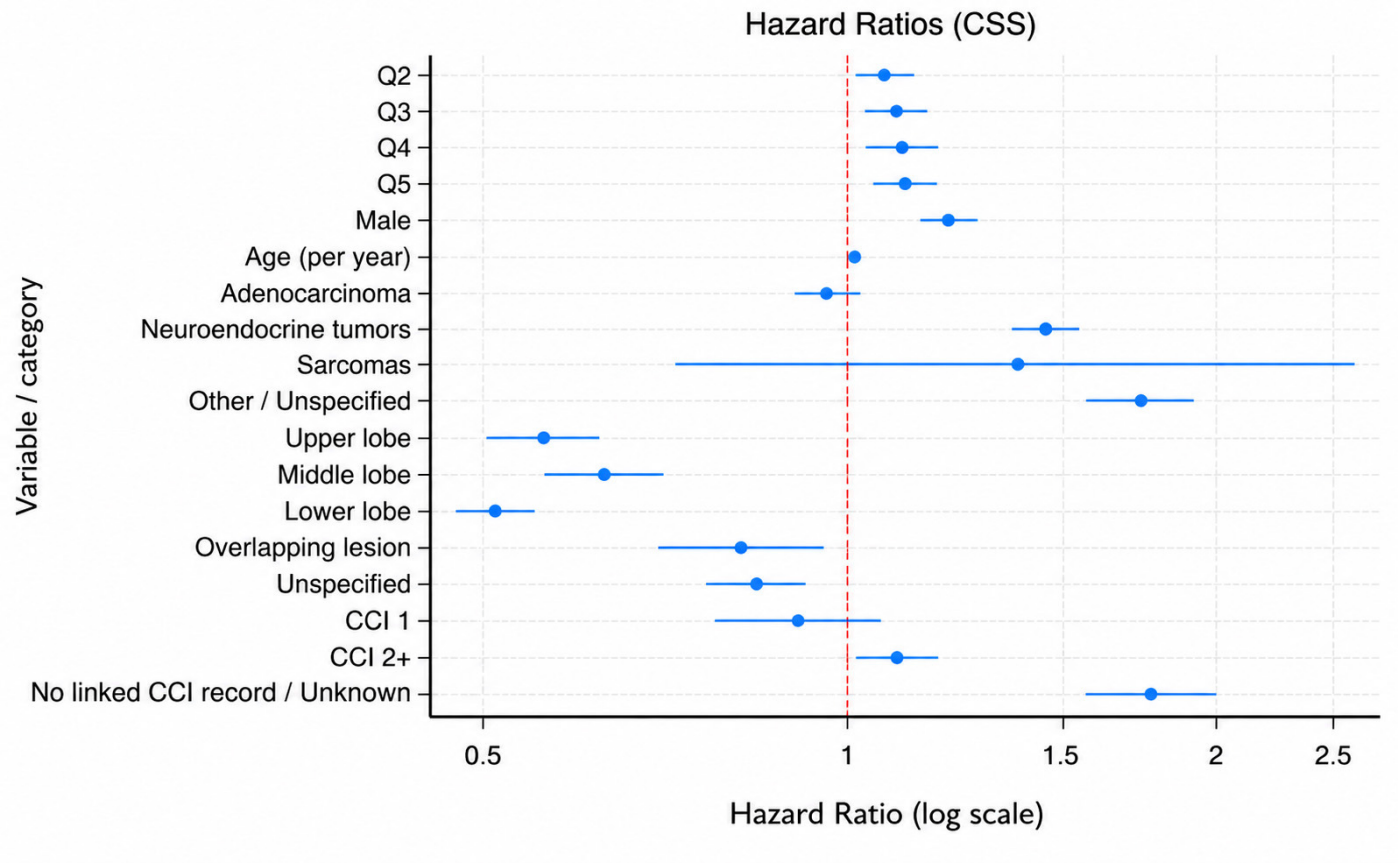
Forest plot of hazard ratios from the primary multivariable Cox model for cause-specific survival. The dashed line indicates HR = 1.

Among the 8,514 deaths recorded in the final analytical cohort, 6,990 were classified as lung-cancer-specific deaths according to the predefined coding algorithm and 1,524 were classified as deaths from other causes and treated as competing events in the Fine–Gray analysis. Thus, all recorded deaths in the final analytical cohort were assigned to either the cause-specific or competing-event category for the survival analyses. Compared with Q1, the sHRs were 1.072 for Q2 (95% CI: 1.016–1.131; *p* = 0.011), 1.022 for Q3 (95% CI: 0.960–1.089; *p* = 0.491), 1.084 for Q4 (95% CI: 1.016–1.156; *p* = 0.015), and 1.097 for Q5 (95% CI: 1.048–1.148; *p* < 0.001). Thus, the most deprived quintile remained associated with a higher cumulative incidence of cause-specific mortality in the presence of competing deaths. The Fine–Gray estimates were broadly directionally consistent with the cause-specific Cox findings, although the two models estimate different quantities and their effect estimates should not be compared as if they represented the same estimand. Complete numerical estimates from the Fine–Gray sensitivity analysis are reported in Supplementary Table S10. Figure 3 provides the corresponding graphical representation of cumulative incidence for the extreme deprivation quintiles.

**Figure 3:**
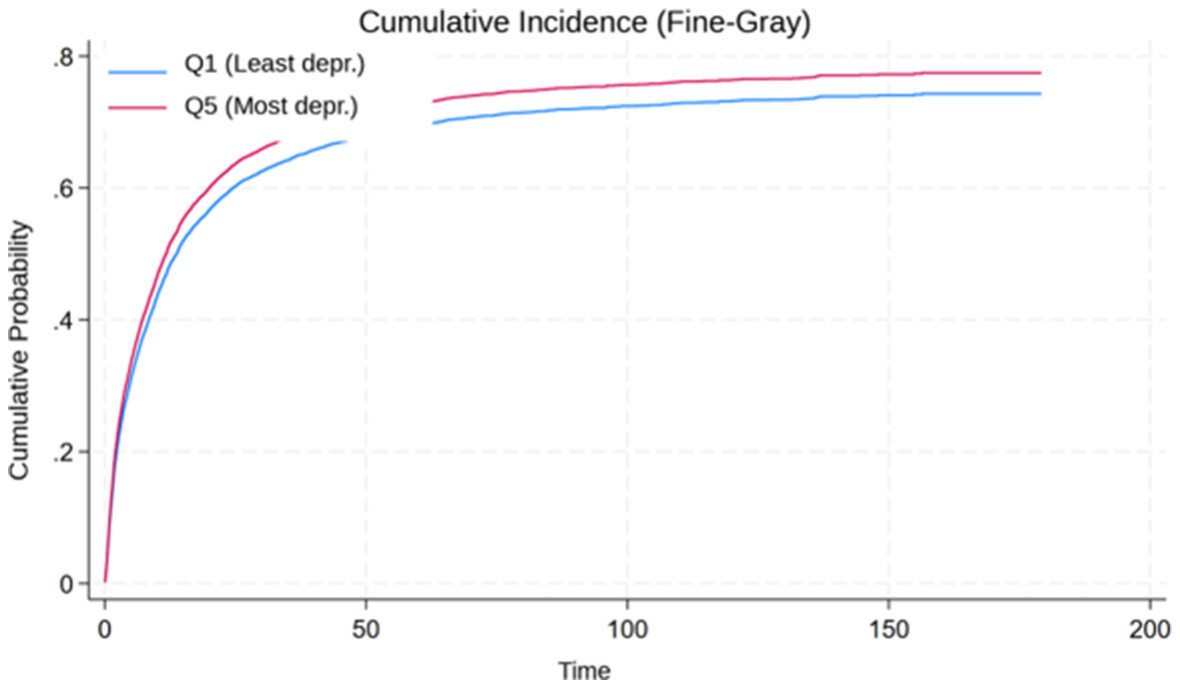
Cumulative incidence of cause-specific mortality according to the Fine–Gray competing-risk model for the extreme deprivation quintiles: Q1 (least deprived) and Q5 (most deprived).

The exploratory deprivation-by-sex interaction model provided no evidence of marked effect modification by sex. With female sex and Q1 as the reference categories, the interaction coefficients on the log-hazard scale were β = −0.018 for Q2 × male, β = −0.070 for Q3 × male, β = 0.034 for Q4 × male, and β = 0.014 for Q5 × male. These corresponded to interaction HRs of 0.982 for Q2 × male (95% CI: 0.869–1.111; *p* = 0.774), 0.933 for Q3 × male (95% CI: 0.760–1.145; *p* = 0.505), 1.034 for Q4 × male (95% CI: 0.915–1.169; *p* = 0.592), and 1.014 for Q5 × male (95% CI: 0.840–1.225; *p* = 0.883). None of the individual interaction terms was statistically significant. A joint omnibus Wald test of the four interaction terms was also non-significant (χ^2^ = 2.45, 4 *df* ; *p* = 0.653). Thus, neither the individual product terms nor the global test provided evidence of effect modification by sex; the interaction analysis remains exploratory. In this parameterization, the main IDISE coefficients estimate the deprivation associations among females, whereas each interaction term represents the ratio between the corresponding deprivation HR in males and that in females.

To further explore the interpretation of the observed associations, a causal analysis matrix inspired by the 5 Whys was constructed. This tool was not used to demonstrate causal relationships, but rather to distinguish the directly observed results from possible behavioral, clinical, informational, and organizational mechanisms not measured in the dataset. The complete matrix is presented in the Supplementary material (Supplementary Tool S2, Supplementary Table S4).

### 3.3 Results of the strategic design phase

The simplified qualitative FMEA organized five proposed failure modes for prospective organizational assessment within the prevention-diagnosis-care pathway. These failure modes were derived from the combination of observed epidemiological findings, identified data gaps, and plausible but unmeasured mechanisms; they should not be interpreted as failures empirically demonstrated by the retrospective cohort. Four failure modes were classified as high priority. Within the FMEA, failure to identify high-risk individuals early was considered a hypothetical organizational failure mode potentially associated with late diagnosis and reduced eligibility for effective treatment. This mechanism was not evaluated in the empirical cohort because stage at diagnosis and treatment data were unavailable; it should therefore be regarded as hypothesis-generating and not as an explanation demonstrated by the observed deprivation association. The lack of structured support for smoking cessation was considered a priority in relation to the geographic distribution of smoking and its potential effects on treatment tolerance, complications, and prognosis.

However, the lack of individual smoking history does not allow for direct verification of this mechanism. Fragmentation across geographic areas, diagnostic services, and oncology was considered within the framework as a plausible organizational mechanism that could contribute to delays, loss to follow-up, or incomplete information; these processes were not directly measured in the present cohort. The frequency of unspecified morphology and the elevated risk observed in the “No linked CCI record/unknown” category were considered signals of data heterogeneity and data-availability issues requiring further investigation; they were not interpreted as evidence of fragmented or less structured care pathways. The failure to record stage and treatment was identified as a high-priority data gap, as it limits the ability to distinguish the contributions of late diagnosis, access to treatment, and treatment equity to differences in survival. Therefore, the definition of a mandatory minimum dataset, accompanied by periodic completeness checks, was proposed. Finally, the use of uniform interventions in heterogeneous regional contexts was classified as a medium-to-high priority. An approach based on proportional universalism was proposed, in which actions are directed at the entire population but with an intensity commensurate with the level of deprivation and regional need. The complete qualitative FMEA is presented in the Supplementary material (Supplementary Tool S3, Supplementary Table S5).

### 3.4 Results of the testing and adaptive execution phase

The output of this phase consists of a PDSA protocol for the future prospective testing of interventions in high-priority DSBs. The cycle includes pilot planning, modular implementation of actions, periodic review of KPIs, and adaptation of interventions based on observed issues. The complete PDSA cycle is presented in the Supplementary material (Supplementary Tool S4, Supplementary Figure S2).

The corresponding Control Plan defines the indicators to be monitored, the data sources, the frequency of data collection, the responsible parties, and the response protocols. No post-intervention results are available, as the pilot was not implemented within the retrospective cohort. The prospective Control Plan is provided in the Supplementary material (Supplementary Tool S4, Supplementary Table S6).

### 3.5 Results of the embedding and sustainability phase

This phase produced an integrated standardization and monitoring framework consisting of a dashboard, a completeness checklist, a training plan, and Kamishibai/LPA audits. The proposed dashboard includes indicators for outcomes, processes, equity, data quality, and sustainability, specifying their source, frequency, responsibility, and use in decision-making. The complete structure of the dashboard is presented in the Supplementary material (Supplementary Tool S5, Supplementary Table S7).

The case closure checklist includes verification of geocoding, IDISE assignment, morphology, topography, stage, treatment, CCI, smoking history, key diagnostic and therapeutic dates, survival status, and cause of death. The presence of missing or inconsistent data triggers a verification and recovery procedure prior to the record's final validation; if the information cannot be found, its unavailability must be explicitly documented. The proposed training plan includes periodic updates on filling out required fields, interpreting indicators, and managing discrepancies. Kamishibai/LPA audits involve concise and repeated checks of data completeness, dashboard updates, and the closure of corrective actions. Since these tools were not implemented during the retrospective period, they constitute project outputs rather than observed sustainability outcomes.

To complement the dashboard, a standardized checklist has been developed for the closure and validation of each case. The purpose of this tool is to verify—before the record is definitively closed—the presence, consistency, and traceability of key demographic, geographic, clinical, and follow-up information. For each element, a compliance criterion and a specific corrective action to be taken in the event of missing, incomplete, or inconsistent data have been defined. The complete checklist is provided in the Supplementary material (Supplementary Tool S5, Supplementary Table S8).

The systematic application of the checklist must be supported by periodic training sessions and verified through Kamishibai/LPA audits, in order to monitor adherence to procedures, promptly identify non-conformities, and verify the completion of the related corrective actions.

### 3.6 Results of the resilience and renewal phase

This phase produced a structured plan for the renewal of the M.A.S.T.E.R. cycle. The plan calls for the periodic updating of data sources, verification of the completeness of variables, monitoring of temporal drift, re-estimation of epidemiological associations, external validation, and an annual strategic review. The structured renewal plan is presented in the Supplementary material (Supplementary Tool S6, Supplementary Table S9).

Updating the data will make it possible to verify whether the geographic distribution of deprivation and the concomitant prevalence of smoking, the composition of the case series, and the survival gradient remain stable over time. The audits will make it possible to monitor, in particular, the availability of data on stage, treatment, smoking history, cause of death, and information derived from record linkage.

The annual strategic review will use this evidence to decide which interventions to maintain, modify, expand, or discontinue, and to formulate a new A3 problem statement, thereby initiating the next iteration of the framework. Since these activities were not implemented during the retrospective period, they represent project governance outputs rather than observed outcomes related to resilience or transferability.

## 4 Discussion

### 4.1 Main results and interpretation

The primary multivariable model showed a modest pattern of progressively higher cause-specific mortality HRs across increasing levels of area-level socioeconomic deprivation. Patients residing in Q5 had an approximately 11% higher cause-specific mortality hazard than those in Q1 (HR 1.11; 95% CI: 1.05–1.18; *p* < 0.001). However, when contextual smoking prevalence from PASSI 2022–2024 was added as a secondary sensitivity adjustment, the deprivation estimates were attenuated and their confidence intervals included unity; for Q5 vs. Q1, the HR decreased to 1.09 (95% CI: 0.99–1.19; *p* = 0.069). Therefore, the strength and statistical precision of the observed deprivation association were sensitive to model specification. However, because PASSI is an ecological measure collected after many of the cancer diagnoses, this attenuation cannot be interpreted as evidence that smoking mediates or explains the association observed in the primary model. The Fine–Gray sensitivity analysis provided complementary evidence: Q5 was associated with a higher subdistribution hazard of cause-specific death compared with Q1 (sHR 1.10; 95% CI: 1.05–1.15; *p* < 0.001). Because the cause-specific Cox HR and the Fine–Gray sHR address different estimands, this concordance should be interpreted as directional robustness rather than as numerical replication of the primary Cox estimate. The direction of the estimates remains consistent with European and international evidence reporting less favorable outcomes in socioeconomically deprived populations (20, 21). The observed associations should not be interpreted as evidence of causality or mediation. The PASSI indicator is aggregated at the regional level and does not represent individual exposure to smoke; moreover, it is not temporally aligned with the entire 2010–2023 cohort. The results therefore point to a possible socio-organizational vulnerability that must be further investigated using individual data, information on clinical pathways, and external validations. The findings should also be interpreted in light of the long enrollment period. Between 2010 and 2023, lung cancer diagnosis and management evolved substantially, including changes in molecular characterization, targeted therapies, immunotherapy, and organization of care. A sensitivity model including diagnostic period (2010–2014, 2015–2019, and 2020–2023) yielded essentially unchanged deprivation estimates. However, categorical period adjustment cannot fully capture the substantial secular changes in diagnosis and treatment, and residual confounding by calendar-time changes therefore remains possible.

### 4.2 Methodological contribution of the M.A.S.T.E.R. framework

The methodological contribution of this study lies in having organized the integration of the Cancer Registry, IDISE, SDO, and PASSI into an explicit process that links context understanding, epidemiological analysis, intervention design, prospective testing, standardization, and cycle renewal. The A3 problem statement and the SIPOC chart clarified the problem, its boundaries, and the information architecture; the Pareto chart, the causal analysis inspired by the 5 Whys, and epidemiological modeling identified the main associations and critical issues; the qualitative FMEA used the observed findings, data gaps, and hypothesis-generating mechanisms to structure proposed failure modes and potential strategic actions; the PDSA and Control Plan defined future prospective testing; dashboards, checklists, training, and Kamishibai/LPA audits outlined the tools for standardization; finally, data updates, drift analysis, external validation, and annual reviews set the stage for a subsequent iteration of the cycle.

In this study, the application of the framework can improve the transparency and reproducibility of the transition from evidence to organizational actions, especially when comprehensive individual data are not available. However, its value should not be overestimated: the last four phases produced tools and project protocols, not a demonstration of improved outcomes. The utility of M.A.S.T.E.R. in this specific context will need to be evaluated through prospective implementation, pre-post or controlled comparisons, stakeholder involvement, sustainability analysis, and multicenter validation.

### 4.3 Smoking as a contextual indicator and public health lever

The smoking prevalence derived from PASSI does not replace individual medical history, but serves as an indicator of the local context and social vulnerability. Its inclusion in the secondary sensitivity model made it possible to account for geographic variation in smoking prevalence, but it does not allow estimation of individual exposure or demonstration of a confounding or mediating role. Because PASSI estimates refer to 2022–2024, they cannot be assumed to represent district-level smoking prevalence at the time of diagnosis for patients diagnosed earlier in the 2010–2023 cohort. Accordingly, the PASSI covariate should be interpreted as an exploratory contextual adjustment rather than as a historical exposure measure. Its lack of statistical association with cause-specific mortality does not exclude an important role of individual smoking behavior in lung cancer prognosis or socioeconomic inequalities. The continued public health relevance of smoking is supported by evidence showing higher prevalence, lower cessation rates, and greater dependence among socioeconomically disadvantaged groups (22, 23). Smoking can also affect prognosis after diagnosis, reducing tolerance to treatments, increasing complications, and worsening survival; quitting shortly before diagnosis, on the other hand, can improve outcomes (24, 25). In practical terms, integrating data on geographic deprivation with the prevalence of smoking in a given area can help identify high-risk districts that should be prioritized for further investigation and where intensified prevention, cessation, and early diagnosis interventions should be evaluated. However, this prioritization must be confirmed using a prospective baseline and district-specific indicators.

### 4.4 Possible diagnostic and organizational mechanisms

The observed association between greater area-level deprivation and cause-specific mortality may be compatible with several unmeasured mechanisms, including lower health literacy, normalization of symptoms, fatalism, smoking-related stigma, economic and logistical barriers, delayed diagnostic assessment, and discontinuity of care (26, 27). However, none of these mechanisms was directly measured in the present study. In particular, the absence of stage at diagnosis, treatment information, and temporal indicators of the care pathway prevents determination of whether the observed socioeconomic association reflects differences in disease stage, treatment access or selection, continuity of care, or other mechanisms. These explanations should therefore be considered hypothesis-generating. Stage at diagnosis is one of the main determinants of prognosis (28). However, the present dataset does not allow stage-related or treatment-related mechanisms to be evaluated. Likewise, the associations observed for unspecified morphology and the “No linked CCI record/unknown” category should not be interpreted as evidence of delayed diagnosis, clinical frailty, or fragmented care, because these explanations were not directly measured. They identify areas requiring further investigation rather than demonstrated mechanisms of the socioeconomic survival association.

### 4.5 Implications for health planning

The epidemiological findings identify potential areas for further investigation, while the organizational actions proposed below should be regarded as hypothesis-driven strategies generated through the M.A.S.T.E.R. framework rather than interventions directly demonstrated to be necessary or effective by the survival analysis. Within this hypothesis-generating framework, several strategies could be considered for prospective evaluation. First, the feasibility and potential value of combining universal prevention with greater intervention intensity in more deprived areas could be assessed according to the principle of proportional universalism (29). Second, future lung cancer screening programs could evaluate whether geographically informed recruitment improves participation or equity in socioeconomically disadvantaged populations, which may experience greater exposure to risk factors and barriers to screening (30, 31). Third, patient navigation, active outreach, and greater primary-care involvement represent candidate organizational strategies that could be tested prospectively in vulnerable areas. Cancer registry data, deprivation indices, and behavioral surveillance could support the selection of pilot areas and the subsequent monitoring of diagnostic access, participation, data completeness, and equity. M.A.S.T.E.R. proposes an operational sequence through which these candidate actions can be tested, monitored, and adapted prospectively.

### 4.6 Strengths

The main strengths are the population design, the long observation period, the large sample size, the exclusion of records with non-positive survival time from the primary analysis and the corresponding sensitivity analysis retaining these observations with a minimal positive follow-up time, the linkage with an administrative CCI data source for additional characterization of comorbidity burden, the integration of complementary data sources, the use of clustered robust variance for DSB, and the use of competing risk models. The application of the M.A.S.T.E.R. framework also explicitly outlines the tools, outputs, responsibilities, and steps proposed for subsequent prospective implementation.

### 4.7 Limitations of the study

This study has several limitations. First, it was conducted in a single local health authority; the results therefore primarily reflect the socioeconomic, organizational, and healthcare context of ASL Napoli 1 Centro and require multicenter validation before they can be generalized to other regions. Furthermore, the retrospective observational design allows for the identification of associations but does not establish causal relationships or demonstrate a mediating role of the contextual prevalence of smoking. Calendar year or period of diagnosis was not included in the primary model. This is relevant because the 2010–2023 observation period encompasses substantial changes in diagnostic work-up, molecular testing, systemic treatment, targeted therapies, immunotherapy, and care pathways. A sensitivity model additionally adjusted for diagnostic period and produced essentially unchanged deprivation estimates; nevertheless, categorical period adjustment cannot capture all secular changes, so residual confounding by calendar time cannot be excluded.

Relevant clinical variables—including stage at diagnosis, treatment received, performance status, biomarkers, treatment adherence, and duration of the care pathway—were not available. Stage and treatment may partly represent downstream pathways linking socioeconomic disadvantage to survival; therefore, their absence does not simply represent lack of covariate adjustment but, importantly, prevents decomposition of the observed association into differences related to stage, treatment access or selection, continuity of care, and other mechanisms. Other unmeasured clinical characteristics may nevertheless contribute to residual confounding. Information on smoking, derived from PASSI for the 2022–2024 period and applied to the 2010–2023 cohort, represents a regional indicator rather than individual exposure, posing a risk of ecological fallacy and temporal misclassification. Historical time-aligned deprivation and smoking measures were not available in the analytical dataset; consequently, the magnitude and direction of any bias arising from this temporal mismatch cannot be fully quantified in the present study. A sensitivity analysis restricted to diagnoses in 2019–2023 was performed. The deprivation point estimates remained above unity, but confidence intervals widened and Q5 was not statistically significant, underscoring the reduced precision of the shorter-period analysis and the continuing need for truly contemporaneous socioeconomic and behavioral measures. The deprivation estimates should accordingly be interpreted as associations with a single-time-point area-level socioeconomic classification rather than with contemporaneously measured socioeconomic exposure at diagnosis. Residence was assigned at the time of cancer diagnosis using historical municipal-registry information, improving the temporal accuracy of geographic assignment. However, longitudinal residential trajectories were not modeled. Residential mobility may therefore have weakened the correspondence between the assigned area-level socioeconomic classification and the socioeconomic context experienced by individual patients over time, potentially introducing additional exposure misclassification. In addition, contextual smoking prevalence was modeled linearly per percentage-point increase, and nonlinearity was not formally assessed. Therefore, nonlinear or threshold relationships between district-level smoking prevalence and cause-specific mortality cannot be excluded. Comorbidity adjustment was based on a precomputed CCI dataset derived from Hospital Discharge Records available for 2014–2023. Because the study-level analytical file did not include the underlying hospitalization dates or the patient-specific look-back period used in the source CCI dataset, the temporal relationship between all diagnoses contributing to the CCI and the date of cancer diagnosis could not be independently reconstructed. This limitation is particularly relevant for patients diagnosed before or shortly after 2014, for whom complete prediagnostic hospitalization ascertainment cannot be assumed from the available study data. Accordingly, the CCI should be interpreted as an administrative proxy for comorbidity burden available through record linkage rather than as a fully validated uniform baseline comorbidity measure across the entire 2010–2023 cohort. A standardized prediagnostic CCI look-back analysis could not be performed because the source CCI dataset did not contain the required patient-specific hospitalization dates. However, a sensitivity model omitting CCI altogether yielded deprivation estimates closely comparable to the primary model, reducing concern that the deprivation association was driven by the uncertain CCI specification. In addition, the “No linked CCI record/unknown” category denotes absence of an available linked CCI record and therefore cannot be interpreted as a defined clinical comorbidity category or as evidence that a patient had no previous hospitalization. Although all recorded deaths in the final analytical cohort could be classified as either cause-specific or competing events using the predefined coding algorithm, the accuracy and source-level completeness of cause-of-death ascertainment could not be independently validated within the present analysis. Misclassification of the recorded cause of death therefore remains possible. The strict definition requiring ICD-10 C33–C34 or, when ICD-10 was unavailable, ICD-9 162 was intended to avoid attributing uncertain or other causes to lung cancer, but this conservative approach may also have classified some uncertain deaths as competing rather than cause-specific events. The primary analysis excluded 666 records with non-positive survival time. Because DCO status could not be separately identified in the analytical dataset used for the survival models, the number of DCO cases and their overlap with non-positive survival time could not be determined separately. Accordingly, these two concepts should not be considered interchangeable. Exclusion of non-positive survival times may preferentially remove patients with very short survival; however, a sensitivity analysis retaining these observations with a minimal follow-up time of 0.1 months produced deprivation estimates closely comparable to those of the primary analysis. From a statistical standpoint, the deprivation estimates were statistically significant in the primary model, whereas they were attenuated and no longer statistically significant after addition of contextual PASSI smoking prevalence. Given the ecological and temporally misaligned nature of PASSI, these differences should be interpreted cautiously. No *a priori* power calculation was performed because this was a retrospective population-based registry study including all eligible cases during the study period. The large analytical cohort (*N* = 10,039) and the high number of cause-specific deaths (*n* = 6,990) provided substantial information for estimation; however, statistical precision should primarily be judged from the confidence intervals rather than from a retrospective power calculation. In particular, the 95% CI for Q5 vs. Q1 in the PASSI-adjusted sensitivity model (0.99–1.19) included both the null value and a modest increase in cause-specific mortality. Therefore, this sensitivity analysis cannot distinguish with certainty between no association and an association of relatively small magnitude. The primary model included only 10 DSB clusters. With a small number of clusters, cluster-robust sandwich variance estimates may provide unstable estimates of uncertainty. Comparison with the corresponding Cox model using conventional model-based variance estimates showed identical point estimates and a broadly consistent deprivation pattern, although inference for Q2 was more sensitive to the variance specification. A finite-sample correction of the cluster-robust covariance matrix with *t*-based inference using 9 degrees of freedom was additionally performed; the Q2–Q5 deprivation coefficients remained statistically significant, although Q2 became borderline (*p* = 0.045). This improves small-cluster inference but does not remove all uncertainty associated with only 10 geographic clusters; multilevel or frailty-based approaches remain useful for future external validation. Schoenfeld-residual diagnostics showed no evidence of non-proportionality for any IDISE term (all *p* > 0.27), but the global diagnostic was significant (*p* < 0.001), driven by departures for several adjustment covariates. Thus, the proportional-hazards assumption appears reasonable for the exposure of primary interest but not uniformly for all nuisance covariates, and the latter coefficients should be interpreted cautiously. Finally, the joint omnibus Wald test for the four IDISE-by-sex interaction terms was non-significant (*p* = 0.653), consistent with the term-specific results and providing no evidence of effect modification by sex.

Finally, the Strategic design, testing and adaptive execution, embedding and sustainability, and resilience and renewal phases of the M.A.S.T.E.R. framework have produced only design tools and protocols, which have not yet been implemented or prospectively validated. Pilot studies, structured stakeholder engagement, pre-post or controlled evaluations, and multicenter validations are therefore necessary to verify their effectiveness, sustainability, and transferability.

## 5 Conclusions

In this population-based cohort within ASL Napoli 1 Centro, greater area-level socioeconomic deprivation was associated with higher cause-specific mortality in the primary multivariable model. Patients residing in the most deprived quintile had an approximately 11% higher cause-specific mortality hazard than those in the least deprived quintile. The association was modestly attenuated and was no longer statistically significant after contextual PASSI smoking prevalence was added in a secondary sensitivity model. Given the ecological nature and temporal mismatch of PASSI with much of the cohort, this attenuation cannot be interpreted as evidence that smoking explains or mediates the association observed in the primary model. Overall, the findings suggest a modest area-level socioeconomic association whose magnitude and statistical precision were sensitive to model specification. They do not establish the clinical or organizational mechanisms underlying the observed pattern. Potential mechanisms involving smoking behavior, stage at diagnosis, treatment access, diagnostic timeliness, or continuity of care remain unmeasured and should be investigated in future studies. On this basis, smoking prevention and cessation, early-diagnosis strategies, patient navigation, improved continuity of care, and strengthened data completeness should be regarded as candidate actions generated by the M.A.S.T.E.R. framework for prospective assessment, rather than as interventions whose effectiveness is demonstrated by the present cohort. The application of the M.A.S.T.E.R. framework has made explicit the transition from epidemiological analysis to organizational design through FMEA, PDSA, control plans, dashboards, checklists, and a periodic review plan. However, these tools represent design outputs and do not demonstrate the effectiveness of interventions that have already been implemented. Prospective and multicenter studies, supported by data on disease stage, treatment, individual smoking status, and duration of the care pathway, will be necessary to confirm the observed associations and evaluate the effectiveness, sustainability, and transferability of the proposed actions.

## Data Availability

The data analyzed in this study is subject to the following licenses/restrictions. The individual-level data are held by ASL Napoli 1 Centro and are not publicly available because they originate from routinely collected health databases and are subject to institutional and data-protection restrictions. The authors are not authorized to redistribute the underlying individual-level data. Access may be considered subject to authorization by ASL Napoli 1 Centro and applicable data-protection requirements. Requests to access these datasets should be directed to ASL Napoli 1 Centro.
