# Supplementary material for: Area-level socioeconomic deprivation and cause-specific survival after lung cancer diagnosis: a population-based cohort study in Naples, Italy
Source: Front Public Health. 2026 Oct 6;14:1959445. doi: 10.3389/fpubh.2026.1959445 (PMC13680184; doi:10.3389/fpubh.2026.1959445)
Supplement: Supplementary file 1 [file Table_1.docx]

**SUPPLEMENTARY MATERIAL**

This Supplementary Material compiles additional descriptive analyses and the methodological and operational tools developed as part of the application of the M.A.S.T.E.R. framework to the study of socioeconomic inequalities in cause-specific survival for lung cancer. The contents are organized according to the six phases of the framework and supplement the information provided in the main text, to which readers are referred for a description of the study design, epidemiological methods, results, and their interpretation.

For the Mapping & Contextualization and Analysis & Diagnosis phases, the tools used to represent the information system, stakeholders, case distribution, and key interpretive mechanisms are presented. For the subsequent phases—Strategic Design, Testing & Adaptive Execution, Embedding & Sustainability, and Resilience & Renewal—the planning tools prepared for the future implementation, monitoring, and periodic review of interventions are provided.

These materials are intended to make the process by which epidemiological evidence was translated into organizational priorities, monitoring indicators, and proposals for improvement more transparent, reproducible, and operationally applicable. The tools related to the last four phases should be interpreted as design and governance outputs and not as results of interventions that have already been implemented or prospectively validated.

**S1. Mapping & Contextualization: system and stakeholder mapping**

**Table S1. Output of the SIPOC diagram for the Mapping & Contextualization phase**

| **SIPOC** | Mapped Data Sources |
| --- | --- |
| Suppliers | Cancer Registry; historical municipal registry; ISTAT; SDO data stream; PASSI surveillance. |
| Inputs | Incident cases C33-C34; residence; IDISE 2021; morphology/topography/grade; CCI; survival status and cause of death; smoking prevalence by sex-DSB |
| Process | Record linkage; geocoding; deduplication; exclusion of records with non-positive follow-up time in the primary analysis; sensitivity analysis assigning a minimal positive follow-up time to these records; assignment of IDISE quintiles; follow-up estimation; Cox and Fine-Gray modeling; translation of evidence into candidate organizational priorities |
| Outputs | Integrated analytical dataset; eligible cohort; IDISE quintile assignment; survival estimates; association profiles by deprivation level; evidence for regional planning. |
| Customers | Strategic direction; epidemiology; DSB; primary care; pulmonology/radiology/oncology; prevention services; regional decision-makers |

**Table S2. Mapping of stakeholders involved in the study and in the potential use of the results**

| **Stakeholders** | **Role in the System** | **Interest** | **Influence** | **Contribution or Use of Results** |
| --- | --- | --- | --- | --- |
| Cancer Registry | Production and validation of population-based cancer data | High | High | Case identification, tumor characterization, and follow-up |
| Organizational Epidemiology | Coordination of data integration and analysis | High | High | Epidemiological interpretation and definition of regional priorities |
| Primary Care Districts | Planning and coordination of local interventions | High | High | Identification of priority areas and implementation of local actions |
| Strategic Management | Organizational guidance and resource allocation | High | High | Approval and implementation of interventions |
| Organizational Information Systems | Management and interoperability of information flows | High | High | Data integration, updating, and monitoring |
| General Practitioners | Identification and guidance of at-risk individuals | High | Medium | Prevention, smoking cessation support, and referral to diagnostic pathways |
| Smoking Cessation Services | Secondary prevention and treatment of tobacco dependence | High | Medium | Management of smokers and monitoring of interventions |
| Pulmonology, Radiology, Pathology, and Oncology | Diagnosis, characterization, and treatment of the disease | High | Medium-high | Improvement of diagnostic timeliness and continuity of care |
| Resident Population | Target audience for prevention and early diagnosis initiatives | High | Medium-low | Participation in prevention, cessation, and screening initiatives |

**S2. Analysis & Diagnosis: Pareto and causal analysis**

**Figure S1. Pareto chart of the recorded morphological categories**.

**Table S3. Distribution of tumor morphology and topography according to IDISE 2021 deprivation quintile.**

| **Characteristic** | **Q1 (n=2,584)** | **Q2 (n=2,214)** | **Q3 (n=1,673)** | **Q4 (n=2,151)** | **Q5 (n=1,417)** |
| --- | --- | --- | --- | --- | --- |
| **Morphology/histotype, n (%)** |  |  |  |  |  |
| Squamous cell carcinoma | 404 (15.6) | 358 (16.2) | 267 (16.0) | 351 (16.3) | 268 (18.9) |
| Adenocarcinoma | 1,115 (43.2) | 946 (42.7) | 703 (42.0) | 887 (41.2) | 555 (39.2) |
| Neuroendocrine tumors | 302 (11.7) | 296 (13.4) | 206 (12.3) | 274 (12.7) | 163 (11.5) |
| Sarcoma | 2 (0.1) | 2 (0.1) | 3 (0.2) | 3 (0.1) | 6 (0.4) |
| Other/unspecified | 761 (29.5) | 612 (27.6) | 494 (29.5) | 636 (29.6) | 425 (30.0) |
| **Tumor topography, n (%)** |  |  |  |  |  |
| Trachea/main bronchus | 175 (6.8) | 145 (6.5) | 103 (6.2) | 160 (7.4) | 93 (6.6) |
| Upper lobe | 764 (29.6) | 688 (31.1) | 521 (31.1) | 622 (28.9) | 379 (26.7) |
| Middle lobe | 76 (2.9) | 71 (3.2) | 63 (3.8) | 68 (3.2) | 51 (3.6) |
| Lower lobe | 378 (14.6) | 308 (13.9) | 214 (12.8) | 314 (14.6) | 226 (15.9) |
| Overlapping lesion | 61 (2.4) | 52 (2.3) | 52 (3.1) | 57 (2.6) | 36 (2.5) |
| Unspecified | 1,130 (43.7) | 950 (42.9) | 720 (43.0) | 930 (43.2) | 632 (44.6) |

Values are n (%), with percentages calculated within each deprivation quintile. The table refers to the final analytical cohort included in the primary cause-specific Cox model (N=10,039). Q1 represents the least deprived and Q5 the most deprived areas. IDISE, Socioeconomic Disadvantage Index.

**Table S4. Causal analysis based on the 5 Whys: plausible mechanisms and level of available evidence.**

| **Interpretive Question** | **Evidence/Interpretation** | **Status in this study** |
| --- | --- | --- |
| Why does Q5 tend to indicate a worse prognosis? | Possible concentration of adverse behavioral, clinical, and organizational factors. | The primary Cox model showed progressively higher HR estimates across deprivation quintiles, with a statistically significant higher cause-specific mortality hazard for Q5 versus Q1; the estimates were attenuated and no longer statistically significant after inclusion of contextual smoking prevalence. |
| Why might smoking be a contributing factor? | The prevalence of smoking is socially distributed and may influence risk, treatment adherence, and complications. | Contextual proxy available; individual exposure not available. |
| Why might diagnosis occur later? | Lower health literacy, normalization of symptoms, stigma, fatalism, and logistical barriers may delay access. | Stage not available: plausible but unverified mechanism. |
| Why might the “unspecified” morphology category be associated with higher mortality? | Potential explanations include differences in disease characteristics or diagnostic completeness; these mechanisms were not directly measured. | Association observed; cause not directly identifiable. |
| Why might the “No linked CCI record/unknown” category be associated with mortality? | The association may reflect differences in data availability or other unmeasured characteristics; its clinical meaning cannot be determined from the available data | Elevated HR observed; this category reflects absence of an available linked CCI record and should not be interpreted as a clinical comorbidity category. |

**S3. Strategic Design: qualitative FMEA**

**Table S5. Simplified Qualitative FMEA of the Prevention-Diagnosis-Care Pathway and Proposed Strategic Actions**

| Proposed failure mode | **Possible effect** | **Evidence available in this study** | **Proposed M.A.S.T.E.R. Initiative** | **Priority** |
| --- | --- | --- | --- | --- |
| Failure to identify high-risk individuals early | Possible delayed diagnosis and reduced eligibility for effective treatments | Stage not available; Progressively higher HR point estimates across deprivation quintiles in the primary adjusted model; estimates attenuated after contextual PASSI adjustment. | Geographically targeted recruitment strategies and future screening with low-dose CT | High |
| Lack of systematic support for smoking cessation | Poorer treatment tolerance, more complications, and a less favorable prognosis | No individual smoking history; contextual proxy available | Systematic referral to smoking cessation services and recording of outcomes | High |
| Fragmentation across regions, diagnostic services, and oncology | Delays, loss to follow-up, and incomplete data | Fragmentation was not directly measured. Unspecified morphology and the “No linked CCI record/unknown” category were treated as signals of data heterogeneity or data availability rather than as evidence of fragmented care. | Patient navigation, active outreach, and designation of a care coordinator | High |
| Failure to record disease stage and treatment | Limited interpretability of survival differences and inability to assess treatment equity | Structural limitation of the dataset | Introduction of a mandatory minimum dataset and periodic completeness audits | High |
| Use of standardized interventions in heterogeneous regions | Reduced ability to address inequalities | Progressively higher HR estimates across deprivation quintiles in the primary model, with attenuation after contextual PASSI adjustment. | Application of proportional universalism and adjustment of care intensity based on need | Medium-high |

**S4. Testing & Adaptive Execution: PDSA and Control Plan**

**Figure S2. Proposed PDSA cycle for adaptive testing of M.A.S.T.E.R. actions.**

**Table S6. Prospective Control Plan for Testing & Adaptive Execution.**

| **KPIs** | **Operational Definition** | **Source** | **Frequency** | **Proposed Manager** | **Proposed Response Policy** |
| --- | --- | --- | --- | --- | --- |
| Stage completeness | Percentage of cases with a valid stage by the time the record is closed | Cancer Registry/Medical Records | Monthly-quarterly | Cancer Registry and Epidemiology | Audit of missing cases and correction of the workflow |
| Treatment completeness | Percentage of cases with documented initial treatment and corresponding date | Oncology, Hospital Discharge Records (SDO), and outpatient data flows | Quarterly | Oncology and Cancer Registry | Additional data linkage and verification of data ownership |
| Documented smoking history | Percentage of cases with recorded status, intensity, and cessation | Primary Care and Oncology | Monthly | Primary Care and Oncology | Training reminder and introduction of required fields |
| Referral to smoking cessation | Percentage of current smokers referred and enrolled | Preventive Care Services | Monthly | DSB and Smoking Cessation Services | Proactive follow-up on unsubmitted cases |
| Time from suspicion to diagnosis | Days between the first documented suspicion and diagnosis | Primary Care, Radiology, and Cancer Registry | Monthly | DSB and Diagnostic Services | Analysis of the causes of cases exceeding the local threshold |
| Time from diagnosis to treatment | Days between diagnosis and first treatment | Oncology, Surgery, and Radiation Therapy | Monthly | Oncology and Case Managers | Escalation of cases exceeding the threshold |
| Adherence to active follow-up calls | Percentage of individuals contacted who complete the proposed action | DSB and Call System | Monthly | DSB and Case Managers | Re-contact and review of communication procedures |
| Loss to follow-up | Percentage of patients without an update by the expected time frame | Cancer Registry and Clinical Services | Monthly-quarterly | Case Managers and Epidemiology | Individual verification and retrieval of missing information |
| Equity of the care pathway | Difference in KPIs between Q1 and Q5 and between DSB | Integrated Dashboard | Quarterly | Epidemiology and Strategic Management | Proportional increase in follow-up intensity for critical DSBs |

# **S5. Embedding & Sustainability: dashboard and validation checklist**

**Table S7. Proposed structure of the Embedding & Sustainability dashboard.**

| **Domain** | **Key Indicators** | **Minimum stratification** | **Source** | **Frequency** | **Manager** | **Action in the Event of Deviations** |
| --- | --- | --- | --- | --- | --- | --- |
| Outcome | CSS; cause-specific mortality; cumulative incidence | Sex, DSB, IDISE | Cancer Registry | Annual | Epidemiology and Cancer Registry | In-depth Analysis of Areas Where Socioeconomic Differences Persist or Worsen |
| Process | Time from suspicion to diagnosis and from diagnosis to treatment; referral for smoking cessation; navigation | DSB, IDISE | Primary Care and Clinical Services | Monthly-Quarterly | Case Managers and Clinical Services | Analysis of Delays and Implementation of Corrective Actions |
| Equity | Absolute and relative differences between Q1 and Q5 | Sex, DSB | Integrated Dashboard | Quarterly-Annual | Epidemiology and Health Services Management | Proportional Increase in the Intensity of Interventions in Critical DSBs |
| Data Quality | Completeness of staging, treatment, smoking status, cause of death, and linkage | Source and producing unit | Cancer Registry and Related Workflows | Monthly-Quarterly | Data Steward | Audit of Incomplete Records, Data Recovery, and Targeted Training |
| Sustainability | Audit coverage; training completed; corrective actions closed | Organizational unit | Quality System | Quarterly | Management and Quality | Review of Responsibilities and a New Audit Cycle |

**Table S8. Proposed checklist for case closure and validation.**

| **Item to Verify** | **Compliance Criteria** | **Action in Case of Non-Compliance** |
| --- | --- | --- |
| Geocoding and DSB | Validated residence and assigned DSB | Verification Against Historical Registry |
| IDISE | Correctly assigned quintile | Review of Territorial Linkage |
| Morphology and Topography | Valid ICD-O-3 codes | Verification Against Pathology Records |
| Stage | Stage available and consistent | Retrieval from Medical Records |
| Treatment | Type and date of first treatment present | Linkage with Oncology, Surgery, or Radiation Therapy |
| CCI | Category assigned or reason for absence | Verification of SDO Data Flow |
| Smoking History | Status and available information recorded | Retrieval from Primary Care or Oncology |
| Key Dates | Dates consistent and chronologically valid | Record Correction |
| Vital Status and Cause of Death | Follow-up updated and cause coded | Verification Against Mortality Data Flows |

**S6. Resilience & Renewal: renewal plan**

The Resilience & Renewal phase defines the periodic activities proposed to reassess data quality, temporal stability, model estimates, external validity, and organizational priorities over time. These activities are prospective governance outputs and were not implemented during the retrospective study period.

**Table S9. Proposed plan for the Resilience & Renewal phase**

| **Renewal activity** | **Proposed frequency** | **Review question** | **Expected output** |
| --- | --- | --- | --- |
| Updating Cancer Registry, PASSI, and SDO data flows and incorporating the most recent available IDISE version | Annually for routinely updated data flows; according to availability for IDISE | Have the geographic distributions, cohort composition, or observed associations changed? | Updated dataset and temporal comparative report |
| Completeness audit | Quarterly to annually | Are stage, treatment, smoking history, cause of death, and record-linkage variables sufficiently complete? | Corrective action plan by data source and responsible unit |
| Drift analysis | Annually or after major changes in data flows | Have variable distributions, data quality, or the magnitude of the observed associations changed? | Drift report and identification of relevant changes |
| Re-estimation of Cox and Fine–Gray models | Annually or after relevant changes | Do the epidemiological associations maintain their direction, magnitude, and interpretability? | Updated estimates and comparison with previous analyses |
| External and multicenter validation | When data from other Local Health Authorities or cancer registries become available | Are the observed deprivation-associated survival patterns and identified priorities reproducible and transferable to other settings? | Multicenter assessment of heterogeneity and external validity |
| Future development of predictive models | After integration of clinical and care-pathway variables | Can individual risk be estimated accurately, with adequate calibration and equity? | Model development and validation protocol |
| Strategic review | Annually | Which interventions should be maintained, modified, expanded, or discontinued? | New A3 problem statement and subsequent M.A.S.T.E.R. iteration |

**Additional statistical results**

**Table S10. Fine–Gray competing-risk sensitivity analysis for cause-specific mortality according to IDISE 2021 deprivation quintile.**

| **IDISE comparison** | **sHR** | **95% CI** | **p-value** |
| --- | --- | --- | --- |
| Q2 vs Q1 | 1.072 | 1.016–1.131 | 0.011 |
| Q3 vs Q1 | 1.022 | 0.960–1.089 | 0.491 |
| Q4 vs Q1 | 1.084 | 1.016–1.156 | 0.015 |
| Q5 vs Q1 | 1.097 | 1.048–1.148 | <0.001 |

Q1 (least deprived) was the reference category. Deaths from causes other than tracheal, lung, or bronchial cancer were treated as competing events. sHR, subdistribution hazard ratio; CI, confidence interval; IDISE, Socioeconomic Disadvantage Index. The Fine–Gray model was performed as a sensitivity analysis and estimates the subdistribution hazard of cause-specific mortality in the presence of competing deaths.

**Additional sensitivity and diagnostic analyses**

**Table S11. Diagnostic-period-adjusted sensitivity analysis for the association between IDISE 2021 and cause-specific mortality.**

| **IDISE comparison** | **HR** | **95% CI** | **p-value** |
| --- | --- | --- | --- |
| Q2 vs Q1 | 1.071 | 1.012–1.135 | 0.018 |
| Q3 vs Q1 | 1.091 | 1.034–1.151 | 0.002 |
| Q4 vs Q1 | 1.103 | 1.027–1.184 | 0.007 |
| Q5 vs Q1 | 1.117 | 1.051–1.187 | <0.001 |

The model used the same covariates as the primary Cox model and additionally included diagnostic period (2010–2014 as reference, 2015–2019, and 2020–2023). DSB-clustered robust variance was used.

**Table S12. Sensitivity analysis restricted to patients diagnosed in 2019–2023.**

| **IDISE comparison** | **HR** | **95% CI** | **p-value** |
| --- | --- | --- | --- |
| Q2 vs Q1 | 1.087 | 0.914–1.293 | 0.345 |
| Q3 vs Q1 | 1.224 | 1.046–1.432 | 0.012 |
| Q4 vs Q1 | 1.237 | 1.031–1.483 | 0.022 |
| Q5 vs Q1 | 1.124 | 0.970–1.303 | 0.121 |

Restricted cohort: n=3,940 patients and 2,297 cause-specific deaths. The model used the same adjustment set as the primary Cox model and DSB-clustered robust variance.

**Table S13. Sensitivity analysis omitting the Charlson Comorbidity Index.**

| **IDISE comparison** | **HR** | **95% CI** | **p-value** |
| --- | --- | --- | --- |
| Q2 vs Q1 | 1.060 | 1.004–1.118 | 0.034 |
| Q3 vs Q1 | 1.093 | 1.022–1.168 | 0.009 |
| Q4 vs Q1 | 1.110 | 1.041–1.185 | 0.002 |
| Q5 vs Q1 | 1.106 | 1.054–1.161 | <0.001 |

The model adjusted for sex, age, morphology/histotype, and tumor topography but omitted CCI/data-availability categories. DSB-clustered robust variance was used.

**Table S14. Finite-sample corrected DSB-clustered inference for IDISE coefficients.**

| **IDISE comparison** | **HR** | **95% CI** | **p-value** |
| --- | --- | --- | --- |
| Q2 vs Q1 | 1.070 | 1.002–1.144 | 0.045 |
| Q3 vs Q1 | 1.096 | 1.025–1.171 | 0.013 |
| Q4 vs Q1 | 1.105 | 1.020–1.198 | 0.020 |
| Q5 vs Q1 | 1.112 | 1.040–1.189 | 0.006 |

The cluster-robust covariance matrix was multiplied by [G/(G−1)]×[(N−1)/(N−p)] and inference used a t distribution with G−1=9 degrees of freedom (G=10 DSB clusters). Point estimates are unchanged from the primary model.

**Table S15. Distribution of patients and events across Primary Health Care District (DSB) clusters.**

| **DSB** | **Patients, n** | **Cause-specific deaths, n** | **All recorded deaths, n** |
| --- | --- | --- | --- |
| 24 | 800 | 533 | 663 |
| 25 | 1024 | 703 | 874 |
| 26 | 1014 | 731 | 887 |
| 27 | 1176 | 803 | 963 |
| 28 | 871 | 621 | 740 |
| 29 | 1009 | 731 | 866 |
| 30 | 877 | 621 | 754 |
| 31 | 1036 | 716 | 880 |
| 32 | 1170 | 822 | 1000 |
| 33 | 1062 | 709 | 887 |

Totals: 10,039 patients, 6,990 cause-specific deaths, and 8,514 all-cause deaths.

**Table S16. Schoenfeld-residual proportional-hazards diagnostics for IDISE terms.**

| **Term** | **p-value** |
| --- | --- |
| Q2 vs Q1 | 0.621 |
| Q3 vs Q1 | 0.334 |
| Q4 vs Q1 | 0.923 |
| Q5 vs Q1 | 0.279 |
| Global model | <0.001 |

Diagnostics were based on the association between Schoenfeld residuals and log follow-up time. No IDISE term showed evidence of non-proportionality (all p>0.27), although the global model diagnostic was significant, indicating time-varying effects for one or more adjustment covariates.
